# Machine learning model for predicting severity prognosis in patients infected with COVID-19: Study protocol from COVID-AI Brasil

**DOI:** 10.1371/journal.pone.0245384

**Published:** 2021-02-01

**Authors:** Flávia Paiva Proença Lobo Lopes, Felipe Campos Kitamura, Gustavo Faibischew Prado, Paulo Eduardo de Aguiar Kuriki, Marcio Ricardo Taveira Garcia, COVID-AI-Brasil

**Affiliations:** 1 Departments of Radiology and Innovation, Diagnósticos da América (Dasa), São Paulo, São Paulo, Brasil; 2 Department of Innovation, Hospital Alemão Oswaldo Cruz, São Paulo, São Paulo, Brasil; Humanitas Clinical and Research Center - IRRCS, ITALY

## Abstract

The new coronavirus, which began to be called SARS-CoV-2, is a single-stranded RNA beta coronavirus, initially identified in Wuhan (Hubei province, China) and currently spreading across six continents causing a considerable harm to patients, with no specific tools until now to provide prognostic outcomes. Thus, the aim of this study is to evaluate possible findings on chest CT of patients with signs and symptoms of respiratory syndromes and positive epidemiological factors for COVID-19 infection and to correlate them with the course of the disease. In this sense, it is also expected to develop specific machine learning algorithm for this purpose, through pulmonary segmentation, which can predict possible prognostic factors, through more accurate results. Our alternative hypothesis is that the machine learning model based on clinical, radiological and epidemiological data will be able to predict the severity prognosis of patients infected with COVID-19. We will perform a multicenter retrospective longitudinal study to obtain a large number of cases in a short period of time, for better study validation. Our convenience sample (at least 20 cases for each outcome) will be collected in each center considering the inclusion and exclusion criteria. We will evaluate patients who enter the hospital with clinical signs and symptoms of acute respiratory syndrome, from March to May 2020. We will include individuals with signs and symptoms of acute respiratory syndrome, with positive epidemiological history for COVID-19, who have performed a chest computed tomography. We will assess chest CT of these patients and to correlate them with the course of the disease. Primary outcomes:1) Time to hospital discharge; 2) Length of stay in the ICU; 3) orotracheal intubation;4) Development of Acute Respiratory Discomfort Syndrome. Secondary outcomes:1) Sepsis; 2) Hypotension or cardiocirculatory dysfunction requiring the prescription of vasopressors or inotropes; 3) Coagulopathy; 4) Acute Myocardial Infarction; 5) Acute Renal Insufficiency; 6) Death. We will use the AUC and F1-score of these algorithms as the main metrics, and we hope to identify algorithms capable of generalizing their results for each specified primary and secondary outcome.

## Introduction

The first coronaviruses discovered in the world were responsible for respiratory and intestinal infections, of which the vast majority had a self-limited course and led mostly to symptoms of common cold. However, they can eventually develop into serious infections in groups at risk (heart diseases, diabetes, among others), in the elderly and also in children. Before the beginning of this current pandemic, two highly pathogenic coronavirus species (SARS and MERS) were described and were responsible for outbreaks of severe acute respiratory syndromes [1]. Regarding this new coronavirus (COVID-19) it was recognized as a causative agent of pneumonia that leads to severe acute respiratory syndrome (SARS-CoV-2). One of its main challenges is its rapid transmission capacity and, in some cases, progression to severe pulmonary conditions that have demanded from the health system a care and combat strategy never seen before in the whole world [1–3].

In Brazil, the expectation is to be of exponential growth, which is why there is a need to implement drastic measures to control population circulation and prevention. Due to the speed of transmission, in most countries, including Brazil, early stage preventive measures were not implemented, causing an explosion of symptomatic cases, many of them severe, with prolonged demand from tertiary health services.

Given this scenario, the inevitable emergence of a large contingent of critically ill patients with COVID-19, with different prognoses, made it crucial to search for early diagnostic mechanisms for better screening and treatment adequacy in each case.

In this context, even before confirmation of the infection, screening for patients with respiratory symptoms is carried out through clinical analysis and imaging tests such as Chest Computed Tomography (CT).

In clinical evaluation, the main described symptoms of infection are fever (88.5%), cough (68.6%), myalgia or fatigue (35.8%), sputum (28, 2%) and dyspnea (21.9%). Other symptoms also described include headache and dizziness (12.1%), diarrhea (4.8%), nausea and vomiting (3.9%). In addition, some hematological changes were observed: lymphocytopenia (64.5%), increased C-reactive protein (CRP) (44.3%), increased lactic dehydrogenase (DHL) (28.3%), and leukopenia (29, 4%) [4].

Chest CT is consider as the best imaging method for assessment of COVID-19, since conventional radiography has low sensitivity, notably in early stages. Typical findings described in the literature include ground-glass opacities (GGO) with a more peripheral distribution, associated with septal thickening and consolidations, usually affecting multiple lobes, although these findings can also be found in other viral pneumonias [5–7].

In this way, machine learning applied to diagnostic imaging can enable the development of tools that can standardize the diagnosis and provide potential findings suggestive of the presence of the disease, its severity, and therefore its prognosis.

Since the beginning of the pandemic, due to its emergency, several studies became available in order to try to predict worst outcomes. Main possible risk factors were evaluated in retrospective studies [7–9]. Most of these studies show advanced age, obesity and other comorbidities (diabetes, severe asthma and other respiratory diseases, heart, liver, neurological and kidney diseases and autoimmune diseases) as the main players for a worst outcome [8, 9].

Machine learning has been used by a growing number of studies in this scenario and in other health related fields, ranging from helping with diagnosis until providing more robust evidence for resource allocation [10, 11]. Adly et al [11], advocated that further studies are need to show all the potential of this tool in clinical practice. The major advantage of using machine learning is that we can combine different variables (demographic and clinical data, laboratory assays and imaging) in a large scale, with reduced rates of misdiagnosis and being able to provide fruitful insights in several aspects of the disease. In our protocol, we will extrapolate the current art by 1) acquiring data from hundreds of patients hospitalized in nine different private and public institutions in Brazil, 2) remove the human error and the high inter-rater agreement on the evaluation of chest CTs, and 3) predict the probability of different outcomes (Time to hospital discharge; length of stay in the ICU; orotracheal intubation due to acute respiratory failure; development of acute respiratory discomfort syndrome, and also other secondary outcomes that will be described later in this protocol).

AI is a comprehensive term of computer science that encompasses a wide variety of subfields and techniques, and is used to describe softwares that perform tasks similar to the human mind, such as "learning" and "problem solving" [9]. Currently, there is a rapid evolution in the capacity of AI solutions, mainly due to the increase in available computing technologies, leading to the emergence of tools with performance comparable to that of humans for complex tasks such as translation, image classification, object detection, object recognition automotive vehicle voice and control [12–16].

Recently, this method of data science has been used in diagnostic imaging through applications in different clinical contexts, such as the detection of radiological findings, intelligent scheduling, reduction of the time to acquire medical images, improvement of the technique and automated decision for clinical support, among others [12, 17].

As the performance of these algorithms is dependent on data used for their training, there is a genuine risk that their execution will be unsatisfactory when applied to data from other institutions [16]. Therefore, for the adoption of these applications, it is necessary to validate their results, through a careful analysis of effectiveness, quality, and safety [18].

The purpose of this study is to validate the results of AI algorithms in the stratification of pulmonary changes in chest CT with multicenter data through scientific methodology to correlate pulmonary involvement with the clinical outcome of patients.

## Hypothesis

The Machine Learning model based on clinical, radiological and epidemiological data will be able to identify imaging and clinical factors correlated to disease severity, and possibly estimate prognosis (hospitalization needs, ICU admission, orotracheal intubation), especially in pandemic times.

## Objectives

### Main objective

Develop a model for stratification of pulmonary changes, using AI algorithms. The model will receive a Chest CT as input and will output the stratification of lung parenchyma, discerning regions of the lung with different densities.

### Secondary objectives

- To evaluate possible changes in Chest CT, through a score, that suggest a worse prognosis in patients with COVID-19, and to identify patterns correlated with worse clinical developments, to guide, in the prospective unfolding of the study, the evaluation of prognostic markers arising automated analyses of Chest CT and contribute to prioritizing treatment according to severity (orotracheal intubation, hospitalization).
- Organize a database with medical images and their respective anonymised reports for CT modality, in different morph-functional changes, in patients with acute respiratory syndromes.
- Evaluate the performance of AI algorithms in this data for tasks such as classification, segmentation, image registration and interpretation of reports.
- Evaluate the impact of the use of these models on medical practice of imaging professionals.

## Materials and methods

We will conduct a retrospectively longitudinal multicenter study (9 Institutions) with at least 160 patients hospitalized from March to May 2020 due to clinical signs and symptoms of acute respiratory syndrome. This study was approved by our National Ethics committee (CONEP) (CAAE: 30548620.0.1001.0008) and approved across each participating center’s ethics committee (Universidade Federal do Rio de Janeiro, Universidade do Estado do Rio de Janeiro, Universidade Federal de São Paulo, Hospital 9 de julho, Hospital São Lucas, Hospital Santa Paula, Hospital Alemão Oswaldo Cruz). CONEP is the central ethics committee. We requested waiver of the consent form due to the retrospective study design.

### Study population

Eligible patients for the study must meet the following characteristics will be considered eligible for the study:

1. Signs and symptoms of acute respiratory syndrome
2. Positive epidemiological history for COVID-19, which may include recent contact (last 14 days) with a confirmed or suspected case, recent trip (last 14 days) to a high-incidence location, or presentation of symptoms after the start of the community transmission phase of SARS-CoV-2 (after 3/20/2020) when the date of hospitalization.
3. Have performed, when symptomatic, a chest computed tomography.

### Exclusion criteria

1. Presence of neoplastic (primary or metastatic) lung lesions, manifested as nodules, masses, consolidations, septal thickening (lymphatic carcinomatosis) or pleural thickening;
2. Chest CT with the presence of movement, acquisition or reconstruction artefacts that make it impossible to apply the segmentation algorithms;
3. CT exams with low quality pulmonary segmentation, or slice thickness greater than 3.0 mm.

### Composition of study outcomes

The definition of the outcomes of this study were:

### Primary outcomes

1. Time to hospital discharge (length of stay, LOS), defined as the period (in days) between the date of admission and the date of discharge (or death);
2. Length of stay in the ICU (ICU LOS), defined as the period (in days) elapsed between admission and discharge (or death) from the ICU;
3. Orotracheal intubation due to acute respiratory failure;
4. Development of acute respiratory discomfort syndrome [19].

### Secondary outcomes

1. Sepsis.
2. Hypotension or cardiocirculatory dysfunction requiring the prescription of vasopressors or inotropes.
3. Coagulopathy.
4. Acute myocardial infarction.
5. Acute renal insufficiency [20].
6. Death.

We will use the AUC and F1-score of these algorithms as the main metrics, and we hope to identify algorithms capable of generalizing their results for each specified primary and secondary outcomes.

**Fig 1:**
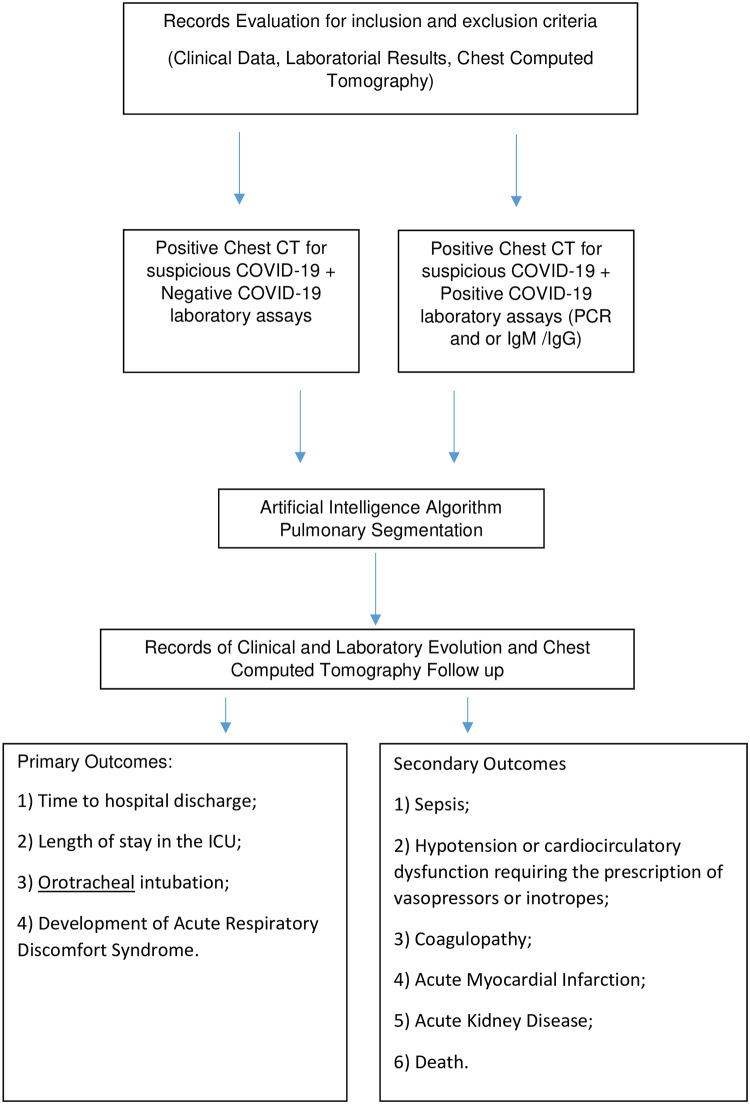
Study flow.

Fig 1 shows the study flow according to the protocol.

### Sample size calculation

In this study, one of the main questions is to verify whether Chest CT is a useful tool as a predictor of severity and worst evolution of COVID-19 pneumonia. Recent studies using a similar methodology [21], showed that the Chest CT scores showed a difference of approximately 17.3 points in the mean value for the scores between the “Survival group” and “Mortality group” among hospitalized patients infected with COVID-19. In this previous study, the pooled standard deviation of the groups, which were approximately 17.5, resulting in an effect size of 0.99. In our study, we would like to know how many patients with “favourable clinical outcome” and with more “severe outcomes” we would need to have. As we expect more patients in the “favourable clinical outcome” group, we made our sample size calculation considering a 3:1 ratio. If the true difference among groups means in our study is 17.3 CT score points, we will need to study at least 15 cases in the “severe outcome” group and 45 control subjects in the “favourable outcome” group to be able to reject the null hypothesis that the population means of both groups are equal with probability (power) of 0,9. The Type I error probability associated with this test of this null hypothesis is 0.05.

### Initial clinical evaluation

- Electronic Collection and Data Management We will develop all forms of data collection in the REDCap system (Vanderbilt University—Tennessee, United States) which is hosted on the server of the participating centers. We will use three main functionalities of this system: a) electronic data collection; b) data management; c) study flow management.
- Demographics and clinical history of patients We will carry out the survey of the history of each participant by consulting the electronic database of each of the participating centers and by reviewing the medical records. We will collect the following information:

* Demographic data: age, gender, race, security number, date of birth, height, weight.

* Clinical data on admission: Onset of symptoms, onset of fever, onset of dyspnea, date of admission, respiratory rate, SOFA score, O_2_ saturation, SAPS-3.

* Patient history: comorbidities (systemic arterial hypertension, diabetes mellitus, asthma, chronic obstructive pulmonary disease, heart disease, dialysis or renal failure, neoplasms, neutropenia, AIDS), in addition to smoking status, and use of medications (notably inhibitors ECA and BRA).

Data will also be analysed such as length of stay in the ICU, in the hospital unit, as well as type of treatment during hospitalization (high oxygen flow, NIV, IOT / invasive ventilation, IOT + extracorporeal membrane oxygenation (ECMO), PaO_2_ / FiO_2_, pronation, haemodialysis).

* Laboratory evaluation:

The following blood tests (complete blood count, creatinine, PCR, D-dimer, IL-6, INR, Troponin, ProBNP, Bicarbonate, Lactate) will be taken from the medical records and identification of IgM and IgG antibodies will be considered for confirmation of the infection-SARS-CoV-2, in addition to nasal and oral swab. Those who have been positive for IgG antibodies since the beginning of the study will already be considered outcome patients, where history data will be thoroughly collected from medical records, especially regarding respiratory symptoms.

* Evaluation by Chest CT:

The Chest CT scans eligible should be performed through volumetric acquisition in a multidetector device, without the use of iodinated contrast medium.

After complete analysis of inclusion criteria, we will classify the exam into one of these standards [22]:

1. Typical COVID-19 pattern:
  - Multiple GGO (also associated or not with consolidations, septal thickening and “reversed halo” opacities); of peripheral location, some with a rounded shape, diffuse predominating in the lower fields.
2. Indeterminate COVID-19 pattern
  - GGO (associated or not with consolidations) of sparse bilateral ill-defined limits, without peripheral predominance and without nodular configuration.
3. Atypical COVID-19 pattern
  In the presence of isolated segmental or lobar consolidation not associated with ground glass, centrilobular micronodules (including the appearance of "tree-in-bud"), cavitation or septal thickening associated with pleural effusion
4. Negative exam
  It will be the absence of infectious lung changes.

The extent grade of pulmonary involvement will be calculated by a tomographic index, where each lung is divided into three regions, namely: upper (above the carina), medium (between the carina and the lower pulmonary veins) and lower (below the lower pulmonary veins). For each region (total of 6 for each patient), a score will be administered, according to the amount of affected parenchyma, being 0 when the entire segment is normal, 1 when the involvement is less than 50%, and 2 when it is greater than 50%. The variation of this index will be from 0 to 12.

A database with medical images acquired at each partner center and their respective reports for the chest computed tomography will be carried out retrospectively, all being anonymised.

### Algorithm development and AI data evaluation

Data set: The data set will consist of Chest CT performed in laboratories belonging to the Coordinator Center network and partner institutions. Radiologists will annotate part of this set of exams, and each annotation will be composed of a polygon that segments the lung in each slice of the computed tomography. Each training instance will then consist of a slice of a computed tomography exam (entry to the model) and the polygon that separates the lung (expected response from the model). It should be noted that the construction of the algorithm will be performed at the Coordinator Center.

The training of the segmentation model will occur in a supervised manner, using the annotated set of data, where each instance of this set must have a polygon that delimits the region referring to the lung in the exam.

We will use the segmentation model to calculate the lung volumes and percentages of GGO and consolidations in the non-annotated Chest CT. This information, along with clinical and laboratorial data, will provide data to develop a machine learning model that predicts the primary and secondary outcomes.

For all models, we will only consider patients that either died or were discharged. For primary and secondary outcomes analysis, we will exclude the unknown cases.

We will compare different classification models, like LightGBM and Catboost. CatBoost is a model from the family of gradient boosting trees. Although powerful, gradient boosting trees tend to overfit the training data when fed with too many features, that is why we restricted the number of features to be studied in this protocol. We will also restrict the depth of the trees to three and fix the learning rate at 0.01 and the patience for early stopping at 50 epochs.

The primary metric is the area under the receiver operating characteristic curve (AUC—ROC).

For each model, we will separate 20% of the considered patients to build our test set. On the other 80%, we will run a 5-fold cross-validation, and all of our results will have a mean and a standard deviation on the holdout patients.

These algorithms will be evaluated using various performance metrics (sensitivity, specificity, positive and negative predictive values, and F1-Score), to determine the generalization of results in our population, and the performance in relation to effectiveness, safety and quality. We will calculate F1 and accuracy for all thresholds, and select the best threshold.

### Missing data

We propose the hypothesis that the machine learning model based on clinical, radiological and epidemiological data will be able to identify imaging and clinical factors correlated to disease severity, and possibly estimate prognosis (hospitalization needs, ICU admission, orotracheal intubation), that is especially needed in pandemic times. In this case, the statistical analysis will consider the intention-to-treat population. Given the design, we expect patients may be were lost to follow-up. Furthermore, we expect a high number of missing data considering that it is a trial conducted with retrospective data collection among hospitalized and discharged patients. We will apply the following strategies to prevent missing data to maintain accuracy of the study:

- Use of a data collection tool that is straightforward and allows the collection of use sufficient data to address the research question.
- Research centers’ data collection monitoring throughout the study.
- Data-monitoring committee to monitor missing data.
- Choose researchers and sites with a good rate of patient with complete follow-up.

We will handle missing data by modern imputation methods as multiple imputation (each missing value will be replaced by a simulated value). The advantages of these methods are that they minimize the loss of precision and power, estimates have almost optimal statistical properties, results are mostly consistent, therefore generally unbiased in large samples with almost perfect efficiency. We will also perform sensitivity analysis to ensure robustness of the primary result testing without and with adjustment for centers.

### Risks

Imaging results will not be identified by name of the patients, therefore it is not possible to characterize the patient, and, also the data will remain with the researchers involved in the project and access to third parties (followers, employers, superiors) will not be allowed, guaranteeing protection against any type of discrimination and/or stigmatization. It is noteworthy that patients with the same conditions have very similar patterns of findings. However, even though remote and despite all precautions, there is always the potential risk of accidental breach of anonymity (loss of reliability). It is noteworthy, however, that the identification of a patient by imaging findings is practically non-existent. In addition, if there is any leak in any patient’s data, all appropriate measures will be taken into consideration, in addition efforts will not be measured to minimize the damage that may occur.

### Benefits

The validation of AI algorithms allows identifying solutions with potential for future use, if approved by the regulatory agencies, in a vast number of conditions. Especially in this pandemic moment, where the identification of prognostic factors can be decisive, in the best conduct and therapeutic strategy not only for patients but also for the entire health system gear at times like this makes this study very valuable and promising.

### Data analysis methodology

#### Statistical analysis

We will use “R” program (version R-3.6.3 for Windows) to perform statistical analyses. Continuous variables will be described by the mean and standard deviation or by the median and interquartile range (IQR) and compared with the t-test, t-test for paired samples, Mann-Whitney or Wilcoxon U test; categorical variables will be expressed as numbers (%) and the CT scores will be compared using the χ2 test or Fisher’s exact test, if appropriate. We assume a bilateral α of less than 0.05 as statistically significant. When calculating samples, a beta error of less than 10% will be used (Power> = 90%).

We will use performance metrics for AI algorithms, such as accuracy, sensitivity, specificity, positive and negative predictive values and F1-Score.

## Expected results

To determine possible findings that predict severity and prognosis with AI algorithms in pulmonary segmentation.

## References

[1] Update on the prevalence and control of novel coronavirus-induced pneumonia as of 24:00 on February 21. https://www.who.int/docs/default-source/coronaviruse/who-china-joint-mission-on-covid-19-final-report.pdf?sfvrsn=fce87f4e_2. Accessed March 17, 2020.

[2] Huang C, Wang Y, Li X, Ren L, Zhao J, Yi H, et al Clinical Features of Patients Infected With 2019 Novel Coronavirus in Wuhan, China. The Lancet. 2020;395(10223): 497–506. 10.1016/S0140-6736(20)30183-5 PMC7159299 31986264

[3] Zhou P, Yang XL, Wang XG, Hu B, Zhang L, Zhang W, et al A pneumonia outbreak associated with a new coronavirus of probable bat origin. Nature. 2020;579(7798): 270–273. 10.1038/s41586-020-2012-7 32015507 PMC7095418

[4] Li LQ, Huang T, Wang YQ, Wang ZP, Liang Y, Huang tb, et al COVID-19 patients’ clinical characteristics, discharge rate, and fatality rate of meta-analysis. J Med Virol. 2020; 92(6): 577–583. 10.1002/jmv.25757 32162702 PMC7228329

[5] ACR Recommendations for the use of Chest Radiography and Computed Tomography (CT) for Suspected COVID-19 Infection. https://www.acr.org/Advocacy-and-Economics/ACR-Position-Statements/Recommendations-for-Chest-Radiography-and-CT-for-Suspected-COVID19-Infection. Accessed April 7, 2020.

[6] Yang R, Li X, Liu H, Zhen Y, Zhang X, Xiong Q, et al. Chest CT severity score: an imaging tool for assessing severe COVID-19. 10.1148/ryct.2020200047. Accessed April 7, 2020. PMC7233443 33778560

[7] Bai HX, Hsieh B, Xiong Z, Halsey K, Choi JW, Tran TML, et al. Performance of radiologists in differentiating COVID-19 from viral pneumonia on chest CT. https://pubs.rsna.org/doi/full/10.1148/radiol.2020200823. Accessed April 7, 2020. 10.1148/radiol.2020200823 PMC7233414 32155105

[8] Williamson EJ, Walker AJ, Bhaskaran K, Bacon S, Bates C, Morton CE, et al Factors associated with COVID-19-related death using OpenSAFELY. Nature 2020;584:430–436. 10.1038/s41586-020-2521-4 32640463 PMC7611074

[9] Zhou F, Yu T, Du R, Fan G, Liu Y, Liu Z, et al Clinical course and risk factors for mortality of adult inpatients with COVID-19 in Wuhan, China: a retrospective cohort study. Lancet. 2020;395(10229): 1054–1062. 10.1016/S0140-6736(20)30566-3 32171076 PMC7270627

[10] Liu F, Zhang Q, Huang C, Shi C, Wang Li, Shi N, et al CT quantification of pneumonia lesions in early days predicts progression to severe illness in a cohort of COVID-19 patients. Theranostics;2020:10(12): 5613–5622. 10.7150/thno.45985 PMC7196293 32373235

[11] Adly SA, Adly AS, Adly MS. Approaches Based on Artificial Intelligence and the Internet of Intelligent Things to Prevent the Spread of COVID-19: Scoping Review. J Med Internet Res;2020;22(8): e19104 10.2196/19104 32584780 PMC7423390

[12] Choy G, Khalizadeh O, Michalski M, Do S, Samir AE, Pianykh OS, et al Current Applications and Future Impact of Machine Learning in Radiology. Radiology.2018; 288: 318–328. 10.1148/radiol.2018171820 29944078 PMC6542626

[13] Mnih V, Kavukcuoglu K, Silver D, Rusu AA, Veness J, Bellemare MG, et al Human-level control through deep reinforcement learning. Nature.2015;518: 529–533. 10.1038/nature14236 25719670

[14] Xiong W, Droppo J, Huang X, Seide F, Seltzer ML, Stolcke A, et al Toward human parity in conversational speech recognition. IEEE/ACM Trans. Audio Speech Language Process. 2017;25: 2410–2423.

[15] Pendleton SD, Andersen H, Du X, Shen X, Meghjani M, Eng YH, et al Perception, planning, control, and coordination for autonomous vehicles. Machines.2017;5: 6.

[16] Nsoesie EO. Evaluating artificial intelligence applications in clinical settings. JAMA Netw Open.2018;1: e182658 10.1001/jamanetworkopen.2018.2658 30646173

[17] Chartrand G, Cheng PM, Vorontsov E, Drozdzal M, Turcotte S, Pal CF, et al Deep Learning: A Primer for Radiologists. RadioGraphics 2017; 37: 2113–2131. 10.1148/rg.2017170077 29131760

[18] Park SH, Han K. Methodologic guide for evaluating clinical performance and effect of artificial intelligence technology for medical diagnosis and prediction. Radiology 2018;286: 800–809. 10.1148/radiol.2017171920 29309734

[19] The ARDS Definition Task Force, Ranieri VM, Rubenfeld GD, Thompson BT, Ferguson ND, Caldwell E, et al Acute Respiratory Distress Syndrome. The Berlin Definition. JAMA.2012;307(23): 2526–2533. 10.1001/jama.2012.5669 22797452

[20] Langham RG, Bellomo R, D’ Intini V, Endre Z, Hickey BB, McGuinness S, et al KHA-CARI guideline: KHA-CARI adaptation of the KDIGO Clinical Practice Guideline for Acute Kidney Injury. Nephrology.2014;19(5): 261–265. 10.1111/nep.12220 24571801

[21] Yuan M, Yin W, Tao Z, Tan W, Hu Y. Association of radiologic findings with mortality of patients infected with 2019 novel coronavirus in Wuhan, China. PLoS One. 2020;15(3):e0230548 10.1371/journal.pone.0230548 32191764 PMC7082074

[22] Simpson S, Kay FU, Abbara S, Bhalla S, Chung JH, Chung M, et al. Radiological Society of North America Expert Consensus Statement on Reporting Chest CT Findings Related to COVID-19. Endorsed by the Society of Thoracic Radiology, the American College of Radiology, and RSNA. 10.1148/ryct.2020200152. Accessed April 1, 2020. PMC7255403 32324653

